# In Vivo Assessment of Shear Wave Propagation in Pennate Muscles Using an Automatic Ultrasound Probe Alignment System

**DOI:** 10.1109/OJEMB.2023.3338090

**Published:** 2023-11-30

**Authors:** Manuela Zimmer, Elsa K. Bunz, Tobias Ehring, Benedikt Kaiser, Annika Kienzlen, Henning Schlüter, Manuel Zürn

**Affiliations:** Institute of Structural Mechanics and Dynamics in Aerospace EngineeringUniversity of Stuttgart 9149 70569 Stuttgart Germany; Institute for Modelling and Simulation of Biomechanical SystemsUniversity of Stuttgart 9149 70569 Stuttgart Germany; Institute of Applied Analysis and Numerical SimulationUniversity of Stuttgart 9149 70569 Stuttgart Germany; Institute of Electrical Energy ConversionUniversity of Stuttgart 9149 70569 Stuttgart Germany; Institute for Control Engineering of Machine Tools and Manufacturing UnitsUniversity of Stuttgart 9149 70174 Stuttgart Germany; Institute for Systems Theory and Automatic ControlUniversity of Stuttgart 9149 70569 Stuttgart Germany

**Keywords:** Automatic ultrasound imaging, shear wave elastography, skeletal muscle mechanics, muscle architecture, gastrocnemius medialis muscle

## Abstract

*Goal:* Skeletal muscle mechanics can be assessed in vivo using shear wave elastography. However, the impact of pennation angle on shear wave velocity (SWV) remains unclear. This study aims to quantify the effect by automatically aligning the ultrasound probe with muscle fiber orientation. *Methods:* We propose an automatic ultrasound probe alignment system and compare it to manual and no alignment. SWV of the gastrocnemius medialis muscle of ten volunteers was measured during rest and isometric contractions. *Results:* The SWV was different between the conditions (*p* = 0.008). The highest SWV was obtained during the automatic alignment and differences between the conditions were most pronounced during high-level contractions. The automatic system yielded more accurate alignment compared to a manual operator (*p* = 0.05). *Conclusions:* The present study indicates that pennation angle affects SWV, hence muscle fiber orientation must be considered to reliably interpret SWV. Using automatic alignment systems allows for more accurate alignment, improving the methodology of ultrasound elastography in skeletal muscles.

## I. Introduction

In vivo characterization of skeletal muscles is crucial for understanding musculoskeletal diseases and improving treatment strategies. For this, the non-invasive ultrasound shear wave elastography (SWE) has recently been shown to represent both passive [1], [2], [3], [4] and active muscle mechanics [5], [6], [7], [8] in healthy cohorts and several studies have demonstrated the clinical potential for SWE, for example for patients diagnosed with myotonia [9], Duchenne muscular dystrophy [10], myasthenia gravis [11] or Parkinson [12]. However, applying SWE on skeletal muscles poses challenges due to the muscles' complex and heterogeneous architecture.

In SWE, a focused ultrasound beam induces shear waves that propagate perpendicular to the imaging plane. Using high frame rate ultrasound imaging, the propagation of shear waves is tracked and the shear wave velocity (SWV) is calculated from the resulting horizontal tissue displacements using image processing [13]. The SWV is displayed for a 2D region of interest (ROI) as a color map overlaid to the conventional B-mode ultrasound image [14]. Assuming transverse isotropic and linear elastic material characteristics, the tissue's stiffness, represented by the shear elastic modulus can be calculated from the SWV [14], [15]. The measured SWV gives insights into muscle stiffness changes. Previous studies have found an increase in muscle stiffness during passive lengthening [1], [2], [3], [4] or muscle contraction [5], [6], [7], [8]. Recent work has shown that changes in SWV may not only be attributed to changes in muscle stiffness but also to changes in tensile loading [16], [17] and that viscoelastic material properties of muscles might have to be taken into account [18], [19].

Moreover, it was shown that the orientation of the ultrasound probe with respect to the muscle's force generation axis significantly affects the measured SWV [19], [20], [21], [22] and that SWV measured with an ultrasound probe oriented parallel to this axis (i.e. longitudinal probe orientation) best represents the muscle's stiffness [20], [23]. For parallel or fusiform muscles that run parallel to the skin surface, placing the ultrasound probe in longitudinal orientation, will lead to an ultrasound image that shows muscle fibers horizontally. The force generation axis of the whole muscle, i.e. the line from proximal to distal tendon, has approximately the same orientation as the force generation axis of individual muscle fibers. Hence, the generated shear waves propagate parallel to the muscle fibers and the measured SWV can be related to muscle mechanical properties along this axis, i.e. the transverse isotropic axis. This is not the case for pennate muscles, whose muscle fibers are at an angle to the longitudinal axis (pennation angle). However, by rotating (i.e. tilting) the ultrasound probe with respect to the skin surface, the muscle fibers in the resulting ultrasound image can be aligned horizontally leading to a shear wave propagation parallel to the muscle fibers. Using simple trigonometry, the SWV in pennate muscles might be underestimated by a factor of cos$(\theta)$ with $\theta$ being the muscle fiber orientation with respect to the horizontal image axis, since SWV is calculated based on displacements along the horizontal image axis and assuming that the generated shear waves follow the fiber orientation [24]. However, shear wave propagation in muscle tissue is complex because of muscles' heterogeneity, anisotropy, finite geometry, and non-zero boundary conditions due to passive and active muscle forces.

Up-to-date only a few studies investigated the effect of pennation angle on SWV in vivo. Although higher SWV in human gastrocnemius medialis muscle (GM) were found when muscle fibers were oriented horizontally compared to no alignment of the ultrasound probe, the authors argue that the differences and effect size were small and concluded that the effect of pennation angle is negligible [24], [25]. For both studies, the percentage difference in SWV was lower than the expected difference based on the cosine-effect [24], [25]. Others did not detect changes in the SWV of cat soleus muscle when rotating the probe up to $15^{\circ }$ [17]. Yet, during muscle contraction, both pennation angle [26], [27], [28] and SWV [5], [6], [7], [8] increase, possibly enhancing the influence of fiber orientation on SWV. So far, no studies investigated the effect of pennation angle on SWV during muscle contraction. Experimental challenges might explain this lack of studies: First, when rotating the ultrasound probe, it must be ensured that the resulting gap between the probe and the skin is filled with gel for proper imaging. Second, due to the increase of pennation angle during muscle contraction, the probe orientation needs to be adapted in real-time. To overcome these challenges, we propose an automatic ultrasound probe alignment system, that fulfills both requirements and test its in vivo use in an experimental study. We investigate the GM of ten volunteers during rest and isometric contraction enabling us to observe the effect of muscle fiber orientation both in passive and active muscle state. We hypothesize that (i) SWV is higher when measured parallel to the muscle fibers (i.e. tilted ultrasound probe rather than no alignment with muscle fibers), (ii) the impact of pennation angle on SWV increases with increased muscle activity, and (iii) our proposed automatic system performs superior compared to a manual ultrasound operator.

## II. Materials and Methods

### A. Participants

Ten healthy volunteers (4 females, 6 males; age: $26.5 \pm 5.4$ years; body weight: $71.4 \pm 9.6$ kg; body height: $174.0 \pm 5.4$ cm) participated in this study after giving written consent. The study has been approved by the ethics committee of the University of Stuttgart (date of approval 22 July 2021, reference number 21-014). All participants were asked to refrain from strenuous or sporting activities 24 hours prior to the study to eliminate possible exercise effects.

### B. Experimental Setup

**Figure 1.**
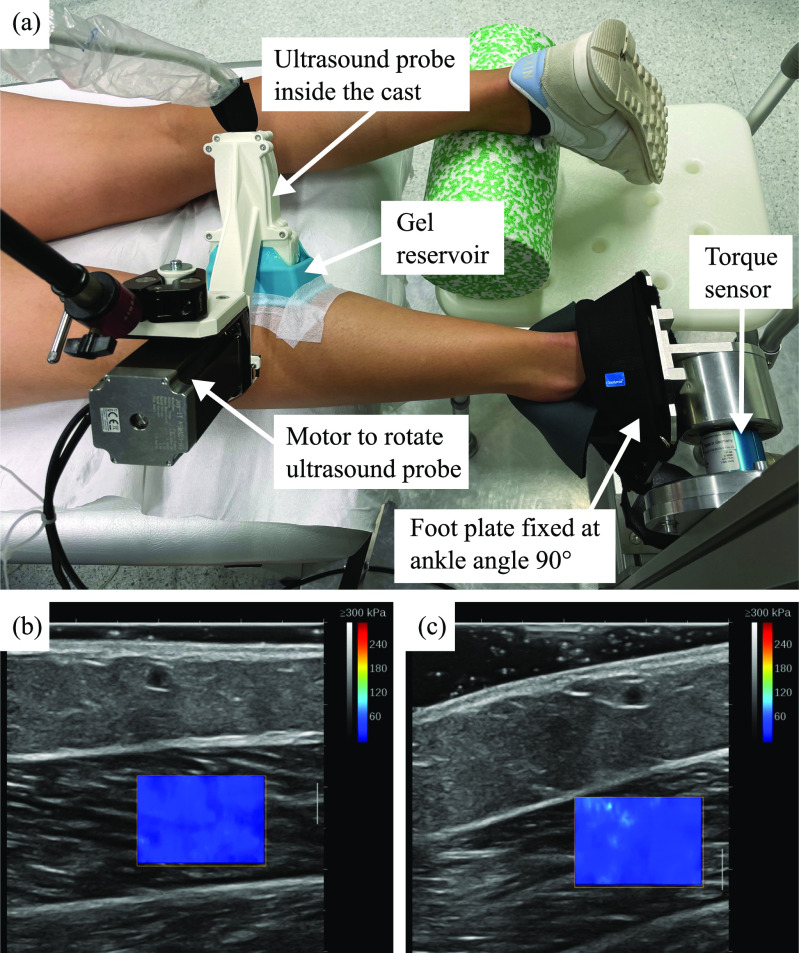
(a) Experimental setup showing a participant during the *automatic aligned* condition. Exemplary ultrasound image during the (b) *not aligned* and (c) *manual aligned* or *automatic aligned* conditions.

Participants lay prone on an examination bed with their knees fully extended. The left foot was strapped to a metal plate with an ankle angle of $90^{\circ }$. The metal plate was part of a custom-made torque measurement system (DF30, 200 Nm, Lorenz Messtechnik GmbH, Aldorf, Germany), that allowed measuring the ankle torque (Fig. 1(a)). A data acquisition system (cDAQ-9174, National Instruments, Austin, TX, USA) and a custom MATLAB script [29] were used to acquire and visualize the torque signals (sampling rate: 2 kHz) as well as synchronously record the ultrasound videos (sampling rate: 19 Hz).

The SWV of GM was measured using an ultrasonic scanner with a linear probe (scanner: AixPlorer Mach30, MSK preset; probe: L10-2, 2-10 MHz; Supersonic Imagine, Aix-en-Provence, France). The ultrasound monitor displayed the B-mode image and B-mode image with SWV color map in two columns. The ultrasound probe was placed in longitudinal orientation in the middle of the muscle belly. The location was marked using a skin marker such that the same position was used for all conditions and trials. The ROI (SWV color map) was selected such that it contained muscle tissue only and did not cover aponeurosis or fat tissue. Depending on the size and position of the ROI, the update frequency of the SWV color map, automatically determined by the ultrasound machine, varied between 1–2 Hz. Fig. 1 shows a representative ROI with a height and width of approximately 1 cm and 1.5 cm, respectively.

Three different conditions of ultrasound probe alignment with respect to the muscle fiber orientation were tested:

1) no alignment
2) manual alignment
3) automatic alignment

In the first condition, the operator placed the probe on the skin without any effort of aligning it to the muscle fiber orientation and was instructed to not apply pressure with the probe to the skin (Fig. 1(b)). In the second condition, the ultrasound operator was instructed to rotate the probe without applying pressure to the skin such that the muscle fibers appeared horizontally on the B-mode image. Horizontal alignment was determined visually by the operator. In the third condition, the probe was rotated using the automatic robotic system (Fig. 1(c)).

For conditions 2 and 3, a silicon box^1^^1^We used a silicone mold (approx. 9.5 × 5.5 × 3.5 cm) for soap casting. filled with ultrasound gel was placed face-down on the skin as a gel reservoir (Fig. 1(a)). The probe was inserted via a slit in the bottom of the box, allowing free rotation without creating an air gap between probe and skin in the reservoir. It was placed in a distance to the skin such that the probe did not touch the skin even during rotation up to $45^{\circ }$. The gel reservoir enabled proper transmission of ultrasound waves during the rotation of the probe. For both conditions, the ultrasound probe was aligned to the muscle fiber orientation during rest before the trial started and the alignment was adapted throughout the trial either manually by the operator or using the automatic setup.

### C. Automatic Ultrasound Measurements

**Figure 2.**
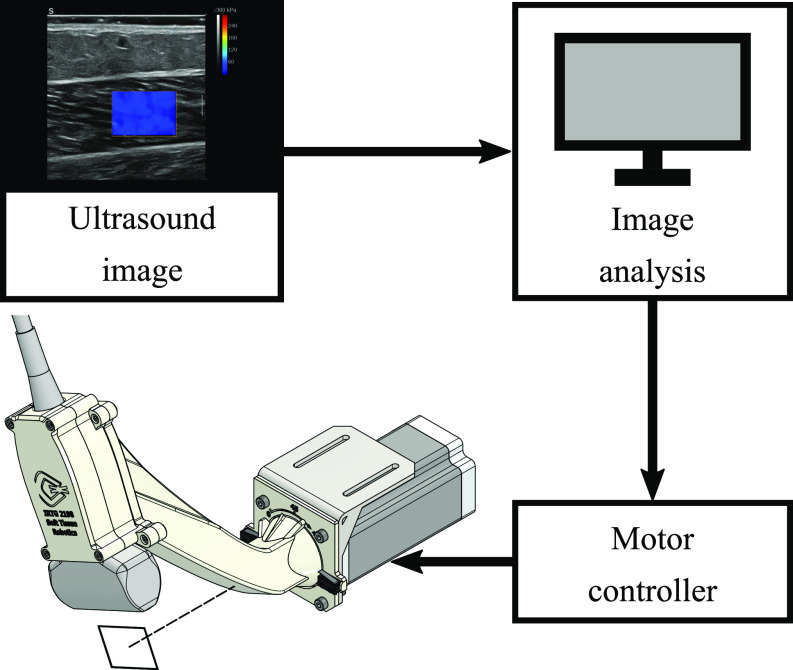
Schematic control cycle of the automatic ultrasound probe alignment system. The probe is placed in a 3D-printed cast that is connected to the stepper motor, enabling rotation of the probe along the indicated axis (bottom left). The ultrasound image is sent to a computer and the fiber orientation is determined by image analysis (top). The resulting angle is sent to the motor controller (bottom right) which calculates the control signal and sends it to the motor.

Using real-time analysis of the ultrasound image, muscle fiber orientation was estimated from the B-mode ultrasound images and a stepper motor rotated the ultrasound probe to align with the fiber orientation. A tripod next to the examination bed held the motor and the ultrasound probe was placed in an attached 3D-printed casting (Fig. 2). Before starting the experimental trials, the tripod was adjusted in height and orientation such that the ultrasound probe was positioned on the muscle belly as described above. The ultrasound image was processed using OpenCV in Python [30] to derive the fiber orientation with respect to the horizontal image axis based on a probabilistic Hough line transformation. The resulting angle was used to control the stepper motor with the control target of $0^{\circ }$. More details about the automatic probe alignment system are presented in the Supplementary Materials.

### D. Experimental Protocol

For each condition, measurements during rest, maximum voluntary contraction (MVC), and submaximal contraction were performed. All contractions were isometric plantarflexions and all trials were repeated three times. MVC trials and measurements during rest lasted for 5 s. The average maximum ankle torque from the MVC trials was used to normalize the following submaximal contractions. Participants received real-time visual feedback on their ankle torque and were asked to maintain a level of 25%, 50%, or 75% MVC for 7 s. Minimum resting periods of 1 min after MVC trials and 30 s after submaximal contractions were applied to prevent muscle fatigue.

Prior to the described trials, a warm-up protocol consisting of two MVC trials and one submaximal contraction per level was performed to familiarize the participants with the tasks.

### E. Data Analysis and Statistics

The ultrasound videos were processed in MATLAB [29]. Color analysis was performed to extract the average SWV per frame from the ultrasound videos. For each video, the ROI was confirmed manually. If the ROI covered areas outside the muscle, a smaller ROI was selected. Averages over time were calculated whereby for MVC and submaximal contractions, the first two seconds were not considered to reduce the influence of motion artifacts. For each task, the three repetitions were averaged. Two exclusion criteria were applied to ensure the quality of the SWE measurements and used as a measure of the SWE recording quality:

1) For each frame, a minimum of 75% of the pixels must be valid (i.e. colored), otherwise, the frame will be discarded.
2) For each trial, a minimum of 30% of the total frames must be valid (i.e. do not meet the aforementioned criterion), otherwise, the trial will be discarded.

From the B-mode images without color map, the muscle fiber orientation was determined manually by calculating the angle of the muscle fibers with respect to the horizontal image axis. Each video was resampled to 1 Hz and for each frame a line was drawn manually representing the muscle fiber orientation. Then, the absolute angle between the line and the horizontal axis was calculated. Consistent with the SWV analysis, the first two seconds were not considered when calculating the average angle over time for MVC and submaximal contractions. Finally, the three repetitions were averaged. During the manual and automatic conditions, we used this angle as a surrogate for the alignment error.

The maximum ankle torque was extracted from each MVC trial and the average torque was calculated from the three repetitions in each condition.

Statistical analysis was performed using statsmodels in Python [31]. To determine the effect of the probe alignment during the different trials on the SWV, alignment error, and the SWE quality measures, a two-way analysis of variation (ANOVA) was performed [32]. One-way ANOVA was used to test the effect of trial type on muscle fiber orientation. We used a significance level of $\alpha = 0.05$ and the Tukey's Honestly Significant Difference test for post-hoc analysis [33].

## III. Results

In eleven out of 150 trials (eight MVC, three 75% MVC trials) the automatic alignment failed, as the ROI was not placed sufficiently during rest. In those cases, during contraction and movement of the muscle, the ROI covered the lower aponeurosis of the GM instead of the muscle fibers and the automatic alignment was performed based on the lower aponeurosis orientation. Those trials were excluded from further analysis.

The quality of the SWE trials defined by the number of valid pixels and the number of valid frames (Table 1) was significantly affected by the trial type ($p < 0.001$ for both) and the alignment condition ($p = 0.017$ for valid pixels, $p = 0.003$ for valid frames). For both quality measures, post-hoc tests located differences between trial types (MVC to passive, 25% MVC, 50% MVC, 75% MVC ($p < 0.001$ for all), 75% MVC to passive, 25% MVC, 50% MVC ($p < 0.001$ for all)) stating that the SWE quality decreases during high contraction levels (MVC and 75% MVC) compared to the other levels tested. No significant differences were found between alignment conditions ($p > 0.05$ for all). However, the average over trials in Table 1 indicates that both quality measures were improved during the manual alignment compared to the other conditions tested. No alignment and automatic alignment led to similar outcomes.

**TABLE I:** Number of Valid Pixels and Valid Frames (In $\%$) During Shear Wave Elastography Color Analysis for Trials and Alignment Conditions Without Excluding Any Trials

	Pixels (%)			Frames (%)		
alignment$\rightarrow$	no	manual	automatic	no	manual	automatic
trial $\downarrow$						
Passive	97.53	99.57	96.15	98.50	100.00	96.69
25% MVC	97.55	98.38	85.80	98.50	99.67	78.43
50% MVC	87.64	97.13	80.92	81.70	98.08	67.80
75% MVC	54.38	75.88	65.03	35.40	66.44	51.66
MVC	27.28	44.17	40.09	5.67	23.89	27.41
trial mean	72.88	83.03	73.60	65.96	83.00	64.40
MVC: maximum voluntary contraction						

After applying the exclusion criteria, the sample size of SWE measurements during MVC was 4 (no alignment), 6 (manual alignment), and 4 (automatic alignment). During 75% MVC, the sample size was 7, 8, and 8, respectively. For all other trials and conditions, a full sample size of 10 was achieved. Due to the small sample size during MVC, the MVC trials were not included in the statistical tests on SWV.

**Figure 3.**
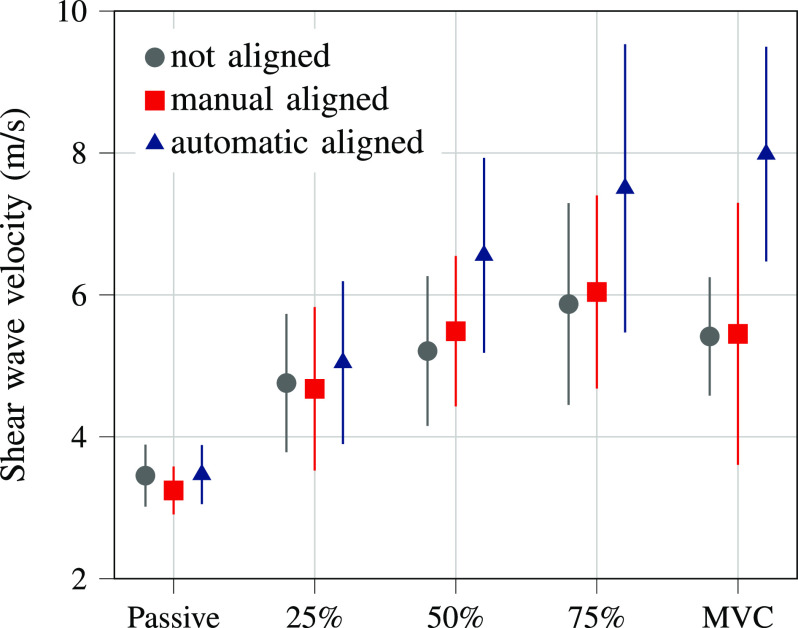
Shear wave velocity of the gastrocnemius medialis muscle measured during rest (passive state), submaximal contractions (25%, 50%, 75% maximum voluntary contraction (MVC)), and MVC. Error bars visualize the standard deviation.

The SWV was significantly affected by trial type ($p < 0.001$) and alignment condition ($p = 0.008$, Fig. 3). Post-hoc tests located the differences between passive and 25% MVC, 50% MVC, 75% MVC ($p < 0.001$ for all) and between 25% MVC and 50% MVC ($p = 0.024$), 75% MVC ($p < 0.001$), demonstrating an increase in SWV with contraction intensity. No significant differences were found between alignment conditions ($p > 0.05$ for all). Fig. 3 suggests that the SWV during automatic alignment was higher compared to the other conditions tested, whereas manual alignment led to similar SWV as no alignment. The muscle fiber orientation during the not aligned condition significantly differed between the trial types ($p < 0.001$) with post-hoc differences between MVC and passive ($p < 0.001$), 25% MVC ($p = 0.001$), 50% MVC ($p = 0.010$), revealing an increased pennation angle during MVC compared to the passive state and submaximal contraction levels.

**Figure 4.**
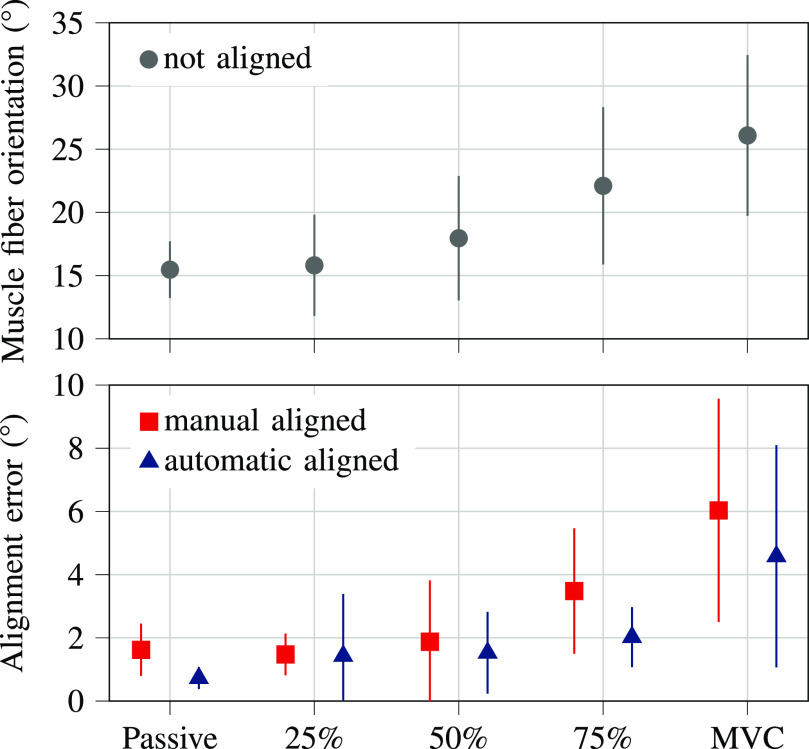
Muscle fiber orientation and alignment error of the gastrocnemius medialis muscle fibers with respect to the horizontal image axis during rest (passive state), submaximal contractions (25%, 50%, 75% maximum voluntary contraction (MVC)), and MVC. Error bars visualize the standard deviation.

The alignment error was significantly affected by trial type ($p < 0.001$) and alignment condition (manual aligned vs. automatic aligned $p = 0.05$, Fig. 4) with the average alignment error in the manual alignment condition ($2.90^\circ \, \pm \,2.69^\circ$) being 1.45 times higher than during the automatic alignment condition ($2.01^\circ \, \pm \, 2.30^\circ$). Post-hoc tests located the differences between MVC and passive ($p < 0.001$), 25% MVC ($p < 0.001$), 50% MVC ($p < 0.001$), 75% MVC ($p = 0.002$), showing higher alignment error during MVC compared to the passive state and submaximal contraction levels.

## IV. Discussion

The ultrasound probe alignment conditions tested in the present study affected the measured SWV of GM significantly, demonstrating the impact of pennation angle on SWV. While we could not locate post-hoc differences between the three conditions, the SWV results (Fig. 3) indicate that SWV might be underestimated in pennate muscles without accurate ultrasound probe orientation as the SWV appears to be higher during automatic alignment of the ultrasound probe. Moreover, the impact of pennation angle on SWV seems to become more prominent with higher muscle contraction.

These findings confirm our first two hypotheses: The SWV is higher when measured parallel to the muscle fibers and the differences amplify with increased muscle activity. We postulate that interference, diffraction, and attenuation of the shear waves are higher when they travel obliquely to the muscle fiber orientation (as in pennate muscles). Hence, the shear waves might get deflected from the assumed perpendicular propagation, and thereby the measured SWV is underestimated [23], [34]. In accordance with previous studies, the effect of muscle fiber orientation on SWV during rest might be negligible as the differences in SWV are small [24], [25]. In active muscle state, it is known that the pennation angle increases with increasing muscle activity [28] and that SWV increases with increasing muscle contraction [5], [6], [7], [8]. From the higher SWV in the aligned conditions during high-level contractions ($\geq 50\%$ MVC) we speculate that during muscle contraction (i) the influence of the muscle fiber orientation on the shear wave propagation is enhanced due to a bigger pennation angle and (ii) faster shear waves are deflected more.

The present results from GM are the first evidence of underestimated SWV in pennate muscles in an active muscle state. Acknowledging that SWV in pennate muscles might be higher than the measured value, can help to interpret and compare SWV between muscles with different fiber architecture. However, it remains an open quest to investigate other pennate muscles to identify more parameters affecting the SWV.

Due to the rotation of the ultrasound probe in the manual and automatic aligned conditions, the ROI is slightly deeper than during no alignment considering that the same muscle fiber region is captured. While some studies found lower SWV with deeper ROI [35], [36], more recent studies could not confirm an effect of the ROI depth on the SWV [37], [38]. While the present study design does not allow to evaluate the effect of ROI depth on SWV, a possible underestimation of SWV due to a deeper ROI would mean that the effect of pennation angle on SWV is even bigger than the present results indicate.

We observed a trend of higher SWV during the automatic compared to the manual condition (Fig. 3). This coincides with the 1.45 times higher angle error during the manual alignment. However, the average absolute difference in angle error is only $0.89^{\circ }$ and thus may not explain the differences in SWV solely. The two conditions differ systematically in how the ultrasound probe is handled and rotated. When the ultrasound probe applies too much pressure on the skin, the muscle tissue can be deformed leading to higher SWV [39], [40]. Exploiting the ultrasound gel reservoir ensured for both conditions that the probe did not apply additional pressure to the skin. Together with the fact that there were no significant differences in the passive SWV between the alignment conditions, we eliminate that probe pressure might have led to higher SWV during the automatic alignment. During isometric contractions, the probe was rotated either manually by the ultrasound operator or by the automatic system. Theoretically, the stepper motor and the mechanical setup can lead to vibration or oscillation of the ultrasound probe, which might affect or interfere with the SWE algorithm that tracks the shear wave propagation. Visually, we could not observe any vibration, shaking, or instability of the probe and to the best of our knowledge, there is no evidence of external vibration or oscillation affecting SWE measurements of scanners similar to the one used in this study.

Besides the influence of ultrasound probe alignment on SWV, the present study also analyzed the SWE data quality by introducing two metrics: The number of valid pixels of the SWV color map for each frame and the number of valid frames per trial. Our results suggest that aligning the ultrasound probe with the muscle fiber orientation improves the data quality of SWE recordings during high-level contractions. It is well known that SWE is sensitive to motion [23] and even during a constant level of muscle contraction, muscles can show some involuntary movements, e.g. shaking or twitching. When the probe is aligned with the muscle fiber orientation, those movements are less noticeable: The fiber orientation remains horizontal during those quick muscle contractions while the fiber orientation changes when the probe is not aligned. While the data quality was improved for all trials in the manual alignment, only high activity levels were improved for the automatic condition. This might be explained by faster or more abrupt automatic movements of the probe that lead to motion artifacts compared to manual adjustment. Moreover, the automatic system might adjust the probe more often due to small changes that are either not observed or are identified as dispensable by a manual operator. A different control law and additional smoothing or adjustment of the motor velocity could improve this.

Finally, the automatic system led to a more accurate probe alignment than the manual alignment, especially during higher activity levels (Fig. 4). While accurate alignment is essential to reliably investigate the influence of pennation angle on SWV, there is also no need for an experienced ultrasound operator when using the automatic system. On the other hand, an automated system leads to additional costs and requires engineering skills to be developed. If there are no resources for a fully automatic ultrasound system, future studies could use a similar ultrasound gel reservoir to enable manual alignment. Moreover, the real-time angle calculation could be incorporated to provide visual feedback to the ultrasound operator. Thereby the operator would be guided by the software to rotate the probe for horizontal fiber orientation instead of using their subjective assessment, possibly improving the manual alignment.

## V. Conclusion

In this study, we developed an automatic ultrasound probe alignment system to overcome the experimental challenge of manually aligning the ultrasound probe to the muscle fiber orientation. We assessed the influence of pennation angle on SWV of GM in ten volunteers both in passive and active muscle states by aligning the ultrasound probe with the pennate muscle fiber orientation and compared it to no alignment. Our results indicate a higher SWV during aligned conditions, most pronounced at high-level isometric muscle contraction. Moreover, the SWE data quality improved during aligned conditions. The automatic system performed more accurately compared to manual alignment, demonstrating the potential of automatic tools to improve ultrasound elastography in skeletal muscles.

Comprehensive datasets on larger cohorts will enable modeling the influence of pennation angle on SWV. Thereby, in the future, a measured SWV can be corrected during post-processing without the need for probe alignment during the examination or standardized probe orientations for SWE of skeletal muscles, especially pennate muscles, can be developed. This will improve the interpretation of the SWV in the context of skeletal muscle mechanics. Automatic systems as proposed in this work will be essential for high-quality data collection.

## VI. Supplementary Material

**Figure 5.**
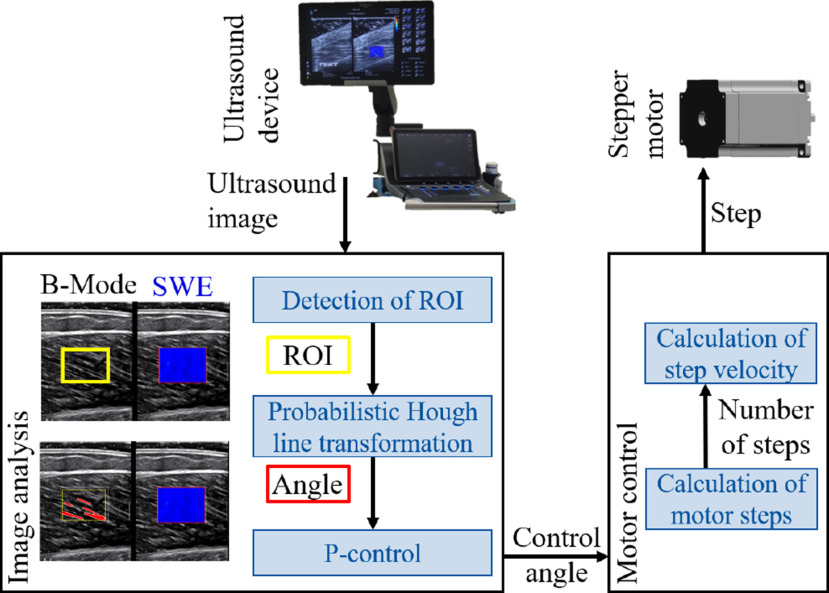
Workflow of the image analysis (left) and motor control (right) used for the automatic ultrasound probe alignment system.

The Supplementary Materials consist of additional results from the experimental study (Table 2, 3) and a detailed description of the proposed automatic ultrasound probe alignment system (Fig. 5).

**TABLE II:** Maximal Dorsiflexion (DF), Maximal Plantarflexion (PF), and Neutral Ankle Angle of All Participants

ID	max. DF ($^\circ$)	max. PF ($^\circ$)	neutral ankle angle ($^\circ$ PF)
1	23	72	37
2	11	50	30
3	15	36	34
4	10	75	45
5	14	65	38
6	11	60	30
7	17	70	35
8	8	65	38
9	8	70	29
10	8	50	25
mean	12.50	64.00	34.10
std	4.59	9.17	5.49

**TABLE III:** Ankle Torque During Maximum Voluntary Contraction Trials of All Participants

ID	no alignment	manual alignment	automatic alignment
1	91.33	109.63	113.37
2	124.60	124.57	154.37
3	160.30	171.73	189.30
4	86.04	85.67	99.23
5	147.47	153.27	133.40
6	180.30	203.50	196.43
7	98.38	95.43	91.43
8	159.40	136.47	135.67
9	66.60	67.52	67.61
10	120.67	122.20	118.17
mean	123.31	127.00	129.90
std	35.72	38.99	39.13

### A. Ankle Angle and Ankle Torque Measurements

To characterize the range of motion of the left ankle, the ankle angles at maximal dorsiflexion (DF), maximal plantarflexion (PF) and neutral position were assessed using a goniometer. Results are presented in Table 2. The MVC ankle torque was not significantly different between the three conditions ($p = 0.060$, Table 3).

### B. Automatic Ultrasound Probe Alignment System

As depicted in Fig. 2, the automatic probe alignment system consisted of (1) a mechanical setup to hold the ultrasound probe and connect it to the motor, (2) image analysis to extract the muscle fiber orientation to determine the control angle and (3) a motor controller.

#### 1) Mechanical Setup

The ultrasound probe was fixed in a custom 3D-printed cast that was connected to the stepper motor (NEMA23-03, 3 Nm holding torque, 200 steps per revolution, Joy-it, SIMAC Electronics GmbH, Neukirchen-Vluyn, Germany). The rotational axis of the motor was defined approximately in the center of the ROI of the ultrasound image, which led to a position 25 mm below the probe. For safety reasons, the motor stopped at a $45^{\circ }$ rotation which was achieved by incorporating two end switches into the cast. The motor was attached rigidly to a commercial camera tripod. The tripod was placed next to the examination bed. Its height and orientation were adjusted for each subject such that the ultrasound probe could be placed properly on the investigated muscle.

#### 2) Image Analysis

The control signal for the motor was calculated via real-time image analysis of the B-mode ultrasound image of the muscle (Fig. 5). The ultrasound images were sent to an external computer using an HDMI grabber (USB3HDCAP, StarTech.com, Hoofddorp, The Netherlands) in order to perform an angle estimation. The image resolution was 640 × 480 pixels. A custom Python script using OpenCV [30] was used to analyze the images automatically.

**Figure 6.**
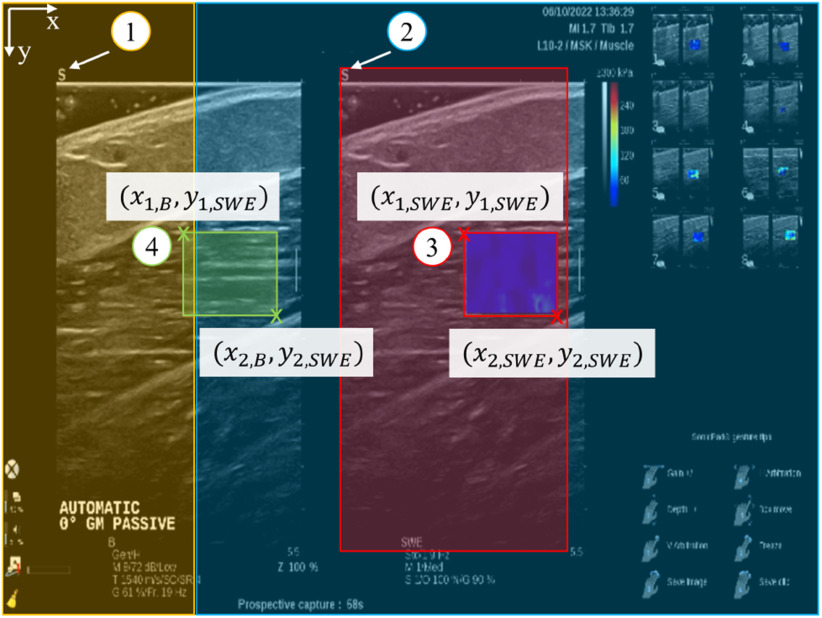
Detection of the region of interest (ROI) in the B-mode image in four steps: Locating the “S” symbol in B-mode (1) and SWE (2) images to calculate the horizontal distance between the two ROI. (3) Detecting the SWE ROI and (4) translating it to the B-mode ROI. The colored overlays represent the cropped images used for these steps.

First, the ROI was located in the B-mode image (Fig. 6). As the ROI does not change within one video, it is sufficient to locate the ROI in the initial frame and use it subsequently. The script automatically located the SWE color map based on the RGB pixel values and then translated the resulting coordinates to the ROI in the B-mode image. The ultrasound machine displayed B-mode and SWE images next to each other, hence, the vertical distance between the two ROI was zero. To determine the horizontal distance $d$, the distinct “S” symbol in the top left corner of the B-mode image and SWE image was automatically located. The colored pixels of the SWE color map were located inside the SWE image by finding all pixels with a standard deviation above 10, i.e. not representing a grayscale color. The minimum and maximum coordinates of the SWE ROI were extracted as $(x_{i,SWE}, y_{i,SWE})$ with $i=\lbrace 1,2\rbrace$ representing the top left and bottom right corners. Finally, the B-mode ROI coordinates $(x_{i,B}, y_{i,B})$ were calculated by $x_{i,B} = x_{i,SWE} - d$ and $y_{i,B} = y_{i,SWE}$.

Using longitudinal ultrasound probe orientation, muscle fibers typically appear as bright lines in B-mode images. These lines were detected inside the extracted ROI by a probabilistic Hough line transformation algorithm. First, a Canny edge detecting filter was applied. The threshold values of the filter were increased iteratively (start value: 50, step size: 10) such that the number of pixels representing edges were less than 10% of the total number of pixels. The edge image was used as an input for the Hough line transformation algorithm, in which the threshold value was decreased iteratively (start value: 100, step size: −1) to find a least ten lines in the ROI. The angles of the lines with respect to the horizontal image axes were calculated and the median angle was used as a representative of the muscle fiber orientation.

The angle was used as a control input in a simple P-controller with proportionality factor $k_{p} = -0.2$. The computer acted as a client and communicated with the motor controller through an Ethernet connection using the middleware ZeroMQ [41] and a conventional client-server configuration sending the control angle.

#### 3) Motor Control

The motor controller (Raspberry Pi 400 with GPIO (General Purpose Input/Output) header, Raspberry Pi OS, Raspberry Pi Ltd, Cambridge, England and Stepper Motor Drivers Board TB6560 with integrated circuit TB6560AHQ, Toshiba Electronics Europe GmbH, Düsseldorf, Germany) was configured as the server and listened for control signals from the client. To initialize the automatic probe alignment system, a reference motion of the motor was performed by rotating the motor across its complete range of motion between the two end switches and counting the number of steps. Thereby the motor position that results in the horizontal orientation of the ultrasound probe was determined without the need for a rotary encoder. After the reference motion, the horizontal position was approached by rotating half of the steps back.

During the experimental trials, the required number of steps was calculated from the control angle using the motor's step size of $1.8^{\circ }$. Less than five steps were directly executed, otherwise the velocity curve was smoothed using an exponential increase and decrease to $v_{\max}$ to avoid jerks. We observed that too high velocities lead to shaking of the mechanical setup. Therefore, we determined $v_{\max}$ from an experimental stability analysis of the mechanical setup and found $v_{\max} = 1.75$ rad/s. The resulting step commands were sent to the stepper motor (Fig. 5).
